# Gemcitabine-oxaliplatin (GEMOX) as salvage treatment in pretreated epithelial ovarian cancer patients

**DOI:** 10.1186/1756-9966-32-49

**Published:** 2013-08-08

**Authors:** Patrizia Vici, Domenico Sergi, Laura Pizzuti, Luciano Mariani, Maria Grazia Arena, Maddalena Barba, Marcello Maugeri-Saccà, Cristina Vincenzoni, Enrico Vizza, Giacomo Corrado, Giancarlo Paoletti, Federica Tomao, Silverio Tomao, Diana Giannarelli, Luigi Di Lauro

**Affiliations:** 1 Division of Medical Oncology B, Regina Elena National Cancer Institute, Rome, Italy; 2 Division of Gynecologic Oncology, Regina Elena National Cancer Institute, Rome, Italy; 3 Division of Medical Oncology, Toraldo Hospital, Tropea, Italy; 4 Department of Medical Oncology-Scientific Direction, Regina Elena National Cancer Institute, Rome, Italy; 5 Department of Gynecologic and Obstetric Sciences, La Sapienza University of Rome, Rome, Italy; 6 Department of Medico-Surgical Sciences and Biotechnologies, Oncology Unit, “SM Goretti” Hospital, “Sapienza” University, Latina, Italy; 7 Biostatistics Unit, Regina Elena National Cancer Institute, Rome, Italy

**Keywords:** Combination therapy, Gemcitabine, Ovarian cancer, Oxaliplatinum, Platinum-resistant

## Abstract

**Background:**

Currently, no clearly superior management strategy exists for recurrent, platinum-resistant ovarian cancer. We tested the efficacy and safety of gemcitabine combined with oxaliplatin (GEMOX) in a multicentre phase II clinical trial.

**Methods:**

Forty one patients with recurrent, platinum-resistant ovarian cancer were enrolled. Prior to study entry, all the participants had received at least one platinum-based regimen. Gemcitabine was administered at 1000 mg/m^2^ as protracted infusion (100 min) on day 1, and oxaliplatin at the dose of 100 mg/m^2^ on day 2 in a 2 hour infusion. Cycles were repeated every two weeks.

**Results:**

We observed an overall response rate of 37% [95% Confidence Interval (CI), 22.3–51.7]. Objective responses plus disease stabilization (clinical benefit) occurred in 78% of patients. Median progression-free survival was 6.8 months (95% CI, 5.8–7.8), and median overall survival was 16.5 months (95% CI, 12.2–20.8). Median time to self-reported symptom relief, which was described by 22 out of 27 symptomatic patients (81.5%), was 4 weeks (range, 2–8). Grade 4 neutropenia and febrile neutropenia were observed in 2 (5%) and 1 (2.5%) patients, while grade 3 anemia was encountered in 2 (5%) patients, respectively. The most common adverse effects of any grade were gastrointestinal symptoms, fatigue and neutropenia. Nine patients (22%) experienced mild allergic reaction to oxaliplatin, with no treatment discontinuation.

**Conclusions:**

In our cohort of recurrent, platinum-resistant ovarian cancer patients, GEMOX showed encouraging activity and manageable toxicity. Under circumstances requiring a rapid disease control, this combination regimen may offer a particularly viable option, particularly in heavily pretreated patients.

## Background

In Western countries, ovarian cancer represents the leading cause of death among women with gynaecological malignancies and the fifth most frequent cause of cancer related death in women [1]. Front-line chemotherapy for advanced epithelial ovarian cancer is currently based on a combination of platinum-derived chemotherapeutic agents (i.e. cisplatin or carboplatin) and paclitaxel. Despite the high response rate and satisfactory median progression-free survival (PFS), over 70% of patients experience disease progression and require further treatments [2].

Re-treatment with a platinum compound in the platinum “sensitive” subgroup, i.e. patients recurring after 12 months from the end of a platinum-based chemotherapy, yields response in up to 70% of cases. Conversely, in platinum “resistant” or “refractory” patients, the administration of agents such as liposomal doxorubicin, topotecan, gemcitabine, vinorelbine, docetaxel, etoposide, ifosfamide, and oxaliplatin, is associated with a response rate ranging from 10 to 33%, with a median PFS of 3–7 months [3,4]. In recent years, patients with platinum-refractory or resistant recurrence have been increasingly treated with more than one line of chemotherapy. However, the actual benefits of currently available treatment options in these patients are poorly documented, particularly beyond the second-line [4,5].

Gemcitabine (GEM; 2,2-difluorodeoxycitidine), a synthetic nucleoside analog of cytidine, inhibits S-phase of cellular cycle. Several trials have confirmed its efficacy in ovarian cancer patients, with response rates up to 22% in platinum-resistant disease and a median response duration ranging from 4 to 10 months. This drug is usually well tolerated, with non-cumulative myelotoxicity being the dose-limiting toxicity [3-5]. Oxaliplatin (OX) is a diaminocyclohexane platinum analog with a partial lack of cross-resistance with carboplatin or cisplatin [6,7]. In recurrent ovarian cancer, OX administration was associated with a 16 to 29% response rate and a substantially different toxicity pattern compared to “classic” platinum compounds [8-11].

The GEMOX combination was first investigated by Faivre et al., showing synergistic effects in human cell lines [12]. A dose-finding combination trial proved feasibility and activity in ovarian cancer patients and phase II trials confirmed its efficacy in recurrent disease, with responses ranging from 9.5% to 37%, median PFS between 4.6 and 7.1 months, and an overall acceptable toxicity [13-17]. The still limited number of studies reporting on treatment outcomes in patients treated with GEMOX, along with the limited evidence concerning the efficacy of this combination in heavily pretreated patients, encourage further research. On this basis, we conducted a multicentre, phase II clinical trial to evaluate efficacy and safety of GEMOX in a cohort of ovarian cancer patients with recurrent, platinum-resistant disease.

## Methods

Patients were eligible if aged 18 years and older and with histologically or cytologically proven, advanced epithelial ovarian cancer. Further requirements were having received at least one previous front-line regimen including paclitaxel combined with carboplatin or cisplatin. Prior radical or debulking surgery, including peritonectomy and Hiperthermic Intraperitoneal Chemotherapy (HIPEC), were allowed. Patient eligibility was also dependent upon the presence of at least one measurable and/or evaluable target lesion documented by imaging, ECOG performance status ≤ 2, adequate bone marrow, cardiac, liver and renal function (glomerular filtration rate according to the Cockroft-Gault formula <60 ml min^-1^), absence of symptomatic brain metastases, peripheral neurotoxicity ≥ grade 1 according to the National Cancer Institute-Common Toxicity Criteria version 4.0 (NCI-CTC v. 4.0), no previous or concomitant serious diseases, including other malignancies except cutaneous basal cell carcinoma and cervical intraepithelial neoplasia. No previous treatment with GEM or OX or any concomitant experimental treatment were allowed.

On study entry, patients were categorized into subsets on the basis of the platinum free interval (PFI), defined as the interval from the last date of platinum dose until progressive disease was documented. Disease was considered as follows: a) *Refractory*, if progression occurred while on the last line of platinum-based therapy or within 4 weeks from the last platinum dose; b) *Resistant*, if the PFI was less than 6 months; c) *Partially platinum-sensitive*, if the PFI was between 6 and 12 months and d) *Fully platinum-sensitive*, if the PFI was longer than 12 months [18]. To our study purposes, we considered eligible all patients but those from the subgroup d.

Disease evaluation included physical examination, weekly complete haemato-biochemical assessment and measurement of serum Ca 125 at every cycle, as well as radiologic evaluation every 3 cycles. All patients received GEM, 1000 mg/m^2^ as protracted infusion (100 min) on day 1, and OX, at the dose of 100 mg/m^2^ administered on day 2 in a 2 hour infusion. Cycles were repeated every two weeks, without prophylactic hematopoietic growth factor administration. Standard antiemetic prophylaxis was administered to all the patients.

Eligible patients who received at least one dose of gemcitabine or oxaliplatin were included in both the efficacy and safety analysis. Efficacy was analyzed for the intention to treat population (ITT), using the enrolled patients as denominator. Tumor response was evaluated according to the response evaluation criteria for solid tumours (RECIST). PFS and overall survival (OS) were calculated from the date of first chemotherapy cycle to the date of disease progression, treatment refusal, death for any cause or lost follow-up evaluation, respectively. Toxicity was graded according to the NCI-CTC v. 4.0. A 25% drug dose-reduction was planned for grade (G) 4 neutropenia or febrile neutropenia, G3-4 thrombocytopenia or other G3-4 extra-hematological toxicities, and G2-3 neurotoxicity; in case of more severe neurotoxicity, treatment discontinuation was planned. G-CSF administration was allowed in case of G4 neutropenia, along with its prophylactic use in subsequent cycles. Chemotherapy was usually administered on an outpatient basis for a maximum of 12 cycles. Treatment was discontinued in case of disease progression, unacceptable toxicity, treatment delay longer than 2 weeks or patient refusal. The study protocol was approved by the Ethic Committee of the Regina Elena National Cancer Institute, the coordinating centre. A written informed consent was obtained from all the enrolled patients prior to any trial procedure. The project was carried out according to the Helsinki Declaration.

### Statistical analysis

Primary objectives of the study were the evaluation of response rate (RR) and PFS, while safety and OS were secondary aims. The optimal Simon's two-stage phase II design was used to determine the sample size [19]. An interim analysis was carried out when the first 13 assessable patients were recruited. If more than 3 responses were observed, 30 additional patients had to be recruited; otherwise, the study had to be terminated. If more than 12 responses were observed in the 43 patients, the regimen was considered sufficiently active with a significance level of 5% and power of 80% to be submitted for further evaluation. The enrolment of 41 patients ensured a sufficient number of events required for statistical analysis. PFS and OS were analyzed according to the Kaplan-Meier method. Follow-up was updated to 30 April 2013.

## Results

### Patients characteristics

Overall, 41 ovarian patients with recurrent, platinum-resistant disease were enrolled between March 2010 and December 2012. Main patient characteristics are listed in Table 1. Median age was 60 years (range, 32–75). Serous adenocarcinomas and poorly differentiated tumours were the most common histological subtypes (24.5%, equally represented), while stage III FIGO at the diagnosis was largely predominant (80%). By preset inclusion criteria, all the patients had received at least one previous platinum-based regimen and were platinum-resistant on study entry. Twenty three patients (56%) were defined platinum-refractory or resistant*,* while for 18 women (44%) the PFI fell in a 6 to 12 month interval (partially platinum-sensitive). Thirty eight patients (93%) had been previously treated with at least two lines of chemotherapy. Eighteen women (44%) had received no less than two previous platinum-based regimens. All the patients had received paclitaxel, one also docetaxel. Thirty seven patients (90%) had also received liposomal doxorubicin.

**Table 1:** Main patient characteristics

**Characteristic**	**No. of patients**	**%**
**Patients evaluable**	41	100
**Age, years**		
Median	60	/
Range	32-75	
**ECOG PS**		
0	13	32
1	21	51
2	7	17
**Tumor histology/citology**		
Serous adenocarcinoma	10	24.5
Adenocarcinoma	7	17
Papillary serous	6	15
Clear cell adenocarcinoma	2	5
Endometrioid	3	7
Mucinous adenocarcinoma	3	7
Poorly differentiated	10	24.5
**Stage at diagnosis**		
I,II	2	5
III (A, B, C)	33 (10, 12, 11)	80
IV	6	15
**N of prior chemotherapy regimens**		
1	3	7
2	12	29
≥3	26	64
**N of prior platinum-based regimens**		
1	23	56
2	9	22
3	9	22

*Abbreviations*: ECOG PS, Eastern Cooperative Oncoloy Group Performance Status.

### Efficacy

A median number of 8 cycles of GEMOX were administered (range, 2 to 12). One patient refused further treatment after the 2nd chemotherapy cycle. All patients were fully evaluable for response and toxicity.

**Figure 1:**
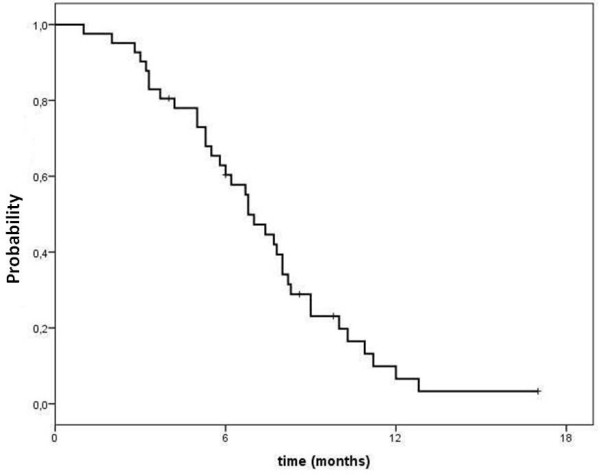
Progression free survival (PFS).

**Figure 2:**
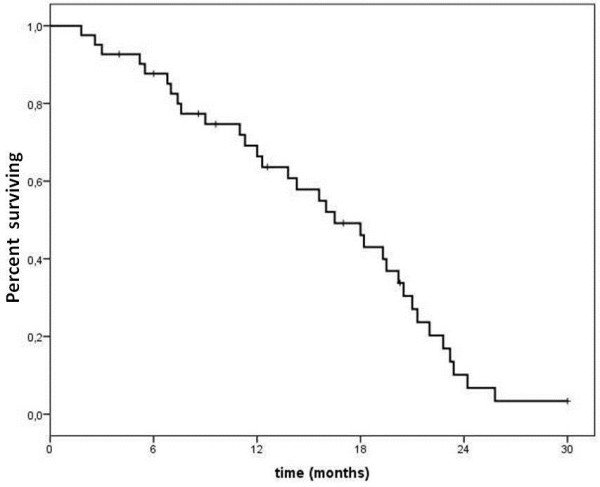
Overall survival (OS).

Based on ITT analysis, 2 (5%) complete responses (CR) and 13 (32%) partial responses (PR) were observed in 41 enrolled patients, for an overall response rate of 37% (95% CI, 22.3 to-51.7%.). Stable disease was observed in 17 patients (41%). A clinical benefit (objective responses + stable disease) was documented in 32 patients (78%) (95% CI, 65–91) (Table 2). Among patients whose disease was originally partially platinum-sensitive, response rate was 50%, while in platinum-resistant or refractory patients response rate was 26%. The PFS was 6.8 months (95% CI, 5.8–7.8) (Figure 1), with no significant difference between initially platinum-sensitive and platinum-resistant patients (7.0 and 6.7 months, respectively). After a median follow-up of 14.5 months (range, 2 to 30), 69.2% and 10.1% patients were alive at 1 and 2 years, respectively; the median OS for the whole cohort was 16.5 months (95% CI, 12.2–20.8) (Figure 2). The median time to self-reported symptom relief, which occurred in 22 out of 27 symptomatic patients (81.5%), was 4 weeks (range, 2–8 weeks); even if symptom improvement translated into objective response in only 8 patients, some degree of amelioration in quality of life was reported by the vast majority of symptomatic patients.

**Table 2:** Objective response in 41 patients

**Responses**	**No. of patients**	**%**
Complete response	2	5
Partial response	13	32
Stable disease	17	41
Progressive disease	9	22
Clinical Benefit	32	78

### Toxicity

The dose-limiting toxicity was hematological, with G4 neutropenia and febrile neutropenia observed in 2 (5%) patients and 1 (2.5%) patient, respectively, requiring G-CSF administration. G1-2 thrombocytopenia were observed in 4 (10%) and 6 (15%) patients, respectively; no cases of G3 or G4 thrombocytopenia were reported. Grade 3 anemia was encountered in 2 (5%) patients, whereas G1-2 anemia was commonly observed (34% and 29%, respectively). Treatment delays because of hematological or extra-hematological toxicities were needed in 4 patients (9.7%). Dose-reductions were required in 3 (7.3%) patients because of G2 neurotoxicity. No cases of G3 or more severe neurotoxicity were observed, while G1 neurotoxicity occurred in 2 patients (5%). Nine patients (22%) experienced mild allergic reaction to OX, usually after the 6th cycle, but no discontinuation of treatment occurred. Nausea and vomiting were mainly G1-2, being G3 in only 2 patients. All patients developed alopecia. No toxic deaths were observed. Main toxicities are reported in Table 3.

**Table 3:** Main toxicity in 41 patients

**Toxicity**	**Grade 1%**	**Grade 2%**	**Grade 3%**	**Grade 4%**
**Hematologic**				
Leukopenia	12	5	5	-
Neutropenia^a^	27	10	10	5
Thrombocytopenia	10	15	-	-
Anemia	34	29	5	-
**Nonhemathologic**				
Nausea/Vomiting	24	15	5	-
Diarrhea	15	15	5	-
Fatigue	29	10	-	-
Neurotoxicity	5	7	-	-
Hypertransaminases	12	10	2.5	-
Conjunctivitis	5	2.5	-	-
Hypersensivity	7	15	-	-

^a^Febrile neutropenia in 1 patient (2.5%).

## Discussion

Recurrent, platinum-resistant ovarian cancer represents a major challenge to modern oncology. GEMOX is a combination regimen with proven activity and overall tolerable toxicity both in pretreated [14-17,20] and first-line treated ovarian patients [21]. However, the related scientific panorama is still remarkably limited by the restricted number of targeted studies and paucity of data on heavily pre-treated patients. In this context, our multicentre, phase II trial provides evidence concerning GEMOX efficacy and safety in a cohort of 41 patients with recurrent, platinum-resistant ovarian cancer. It is noteworthy that among patients included, all but three had received at least two previous lines of chemotherapy.

In our cohort, the GEMOX regimen yielded an overall response rate of 37% (95% CI, 22.3 to-51.7%). In addition, induced objective response plus disease stabilization (clinical benefit) occurred in 78% of patients and relief from disease-related symptoms was reported by the majority of symptomatic patients (81.5%), even though this did seldom translate into objective response. Overall, the regimen was well tolerated, with the major reactions being hematological. The choice of a biweekly schedule instead of a 3-weekly regimen is thought to determine more grade 2–3 peripheral neurotoxicity, while the 3-weekly administration usually gives rise to more severe myelotoxicity. In our study, no significant increase of peripheral neurotoxicity occurred. Indeed, no patients experienced grade 3 neurotoxicity, being neurotoxic effects manageable in the majority of patients.

Results from our trial fairly compare with those from most of the previous reports [14-17,20]. Conversely, due to modest response and relatively high toxicity, Harnett and colleagues defined the GEMOX regimen “unsatisfactory for further study”, but, in this trial, the inclusion of eighteen women (20%) diagnosed with primary peritoneal and Fallopian tube carcinomas, rare tumours commonly associated with the hereditary breast and ovarian cancer syndrome, might have added heterogeneity to the study population and diminished comparability to other studies. Moreover, dissimilarities in the administration schedule might help explain discrepancies in safety outcomes [22].

In the setting of recurrent, platinum-resistant disease, GEM has been variously combined with other drugs. Several trials assessed efficacy and tolerability of GEM/paclitaxel combination, reporting responses in up to 40% of paclitaxel-naïve patients [23]. The combination of GEM/topotecan was tested in phase I-II trials, with some encouraging results even in resistant disease [24], while GEM/docetaxel combination offered response rate of 25% in platinum resistant patients [25]. The GEM/liposomal doxorubicin regimen was used in mostly platinum resistant ovarian cancer patients, yielding response rates ranging from 22 to 42.8%, and a median time to progression and OS from 2.7 to 7.7, and 8.4 to 17 months, respectively [26-31]. Oral etoposide, vinorelbine, irinotecan provide examples of further drugs variously combined with GEM in recurrent, platinum resistant ovarian cancer, with response rates between 10 and 30% [32]. Some authors tested a triple combination including GEM as salvage treatment in resistant disease, without significant benefit over doublets or single-agent [33].

In advanced ovarian cancer, OX was less extensively evaluated compared to GEM. In pretreated patients, OX combination with topotecan and liposomal doxorubicin yielded some encouraging results, showing 29% and 31.5% of responses, with a median PFS and OS of 5.5 to 7.3 and 10 to 15.5 months in mostly, though not exclusively, platinum resistant patients [34-37]. OX-based combinations with paclitaxel or fluorouracil appear promising in platinum resistant disease [38-40]. In this setting, further doublet combinations including docetaxel/irinotecan, carboplatin/irinotecan, and topotecan/etoposide showed results comparable by magnitudo to those of single-agents [41-43].

The potential advantage of combination regimens over single agent therapy in patients with recurrent, platinum resistant disease is still under debate. Indeed, results from several randomized clinical trials consistently favour the use of single agents. However, under circumstances requiring a rapid disease control, particularly in heavily pretreated patients, and with large amount of disease, combination schemes may represent a valid therapeutic option targeted at symptom palliation and eventual objective response, with an acceptable toxicity [44-46]. Based on our results and consistently with previous reports, the GEMOX regimen administered according to the schedule described in the present trial showed encouraging results, given the induction of response or disease stabilization in 78% of cases and relief from symptoms in a even higher percentage of symptomatic patients (about 81%). A comparison of the disease control duration and patient quality of life achieved with GEMOX or single agents will be needed in future studies.

Several molecularly targeted agents have been tested in ovarian cancer, now entering clinical trials. Recently, the Aurelia trial has shown advantages in PFS in the experimental arms, including standard chemotherapy (topotecan, liposomal doxorubicin, or paclitaxel), in combination with bevacizumab, over chemotherapy alone in the setting of platinum resistant patients [47].

A preliminary experience of weekly administration of GEMOX and bevacizumab in recurrent refractory ovarian cancer showed an overall response rate of 32%, with a very high rate of clinical benefit (79%), and a median PFS of 4.5 months, with mild toxicities [48]. Further trials of targeted agents in combination with chemotherapy are ongoing, aiming at the identification of predictive biomarkers and deeper knowledge of molecular biology of ovarian cancer [49]. In the meantime, the choice of “standard” chemotherapy with drugs exhibiting no cross-resistance with platinum, paclitaxel and liposomal anthracyclines, remains a reasonable option in the setting of pretreated and resistant disease. However, at present, no clearly superior management strategy exists for recurrent, platinum resistant/refractory ovarian cancer, particularly in heavily pretreated patients, and beyond the third line, response rates significantly decline, with no reported advantages in OS [3]. In this setting, single-agent therapy is usually recommended, and combination regimens have frequently been shown to increase toxicity without benefit in PFS or OS. Still, given the particularly poor prognosis of pretreated and resistant ovarian cancer patients [50], optimization of quality of life at the lowest toxicity might be a more appropriate outcome compared with survival. In such a context, the GEMOX combination may offer a viable option to patients with recurrent, platinum resistant disease.

## Conclusions

In a cohort of 41 recurrent platinum resistant epithelial ovarian cancer patients, the GEMOX regimen showed encouraging results both in terms of treatment efficacy and manageable toxicity. Moreover, independently on its translation into objective response, self-reported symptom relief was described by the majority of symptomatic patients and occurred in an acceptable time window. On this basis, GEMOX may offer a particularly viable option in this patient population, particularly in heavily pretreated women.

## Competing interests

The authors declare that they have no competing interests.

## Authors’ contributions

LDL and PV conceived and designed the study, DS, LP, LM, MGA, MB, MMS, CV, EV, GC, GP, FT, ST collected and assembled the data, DG performed the statistical analysis, PV wrote the manuscript. All authors read and approved the final manuscript.
